# Anatomical Atlas

**Published:** 1844-04

**Authors:** 


ANATOMICAL ATLAS.

Through Mr. James Maxwell, jr., we have received from the publishers, Messrs. Lea & Blanchard, part second of this beautiful work, containing ninety-one figures illustrative of the dermoid and muscular systems. The execution of this, as of the preceding number, is very admirable, and must commend the Atlas to the profession as one of the first works of its class. It furnishes all the information that drawings can afford, of the structure of the human system. No student, who is trying to acquire a knowledge of anatomy without the aid of subjects, ought to be without it; and the practitioner whose anatomical reminiscences are beginning to fade will find it a valuable remembrancer. Y.

